# Speed-Interactive Pedaling Training Using Smartphone Virtual Reality Application for Stroke Patients: Single-Blinded, Randomized Clinical Trial

**DOI:** 10.3390/brainsci9110295

**Published:** 2019-10-27

**Authors:** Kyeongjin Lee

**Affiliations:** Department of Physical Therapy, College of Health Science, Kyungdong University, Gangwon-do, 24764, Korea; kjlee@kduniv.ac.kr

**Keywords:** stroke, pedaling, virtual reality, trunk, postural balance

## Abstract

This study aimed to investigate the effects of speed-interactive pedaling training (SIPT) using a smartphone virtual reality application to improve lower limb motor function, trunk sitting balance, and gait in stroke patients. Forty-two patients who had previously experienced a stroke and could sit independently participated in the study. The subjects were assigned to the SIPT group (*n* = 21) and the control group (*n* = 21). The SIPT group had cycle training with SIPT for 40 min a day, five days a week, in a six-week period, in addition to conventional therapy. The control group had cycle training without SIPT and conventional therapy. The Fugl–Meyer Assessment, postural sway, modified functional reach test, trunk impairment scale, and spatiotemporal parameters of gait were used to assess the changes in lower extremity function, the static balance of sitting, the dynamic balance of sitting, and gait ability after the intervention. The Fugl–Meyer Assessment, postural sway, modified functional reach test, trunk impairment scale, and gait ability in the SIPT group were significantly better compared to that of the control group (*p* < 0.05). Based on this result, we propose that SIPT, which improves function, balance, and gait, could be used as an effective training method to improve patients’ functional activities in the clinical setting. The results of this study suggest that SIPT could be used as an effective training method to restore a patient’s function by improving trunk balance and motor function.

## 1. Introduction

Strokes are caused by cerebral hemorrhage or infarction, and damage to the cerebral cortex causes various complications such as cognitive, sensory, and motor disorders [1]. Hemiplegia is one of the main complications, and the asymmetry of both sides of the hemiplegic patient’s body leads to loss of function and difficulties in daily life. Paralysis of the upper limbs results in disability of daily activities, and paralysis of the lower limbs causes difficulty in transferring or walking [2,3]. The main goal of rehabilitation in stroke patients is to improve the gait ability to be able to recover and move to the desired location [4].

Gait training is the primary method to improve walking ability, but stationary bicycles are used in preparatory stages or for patients who cannot apply gait training [5,6]. When pedaling, the lower limb muscles are very similar to walking, which helps strengthen these muscles and is effective in training the reciprocal patterns of movement [7,8,9]. In the previous study, biofeedback training, electromyogram feedback training, and virtual reality training were combined to improve the effects of bicycle exercise in stroke patients [5,6,10,11].

Recently, a number of studies have reported the application of advanced technology to rehabilitation. Robots, functional electrical stimulation, and virtual reality are used as new tools for rehabilitation [12,13,14]. Among them, virtual reality is applied to patients who are unable to access various environments due to difficulty in movement, and their effects have been proven [15]. Virtual reality provides the patient with a more realistic, immersive, and improved sensory perception experience and facilitates interesting motor learning based on various feedback mechanisms [16,17].

Virtual reality, which is applied to improve the function of stroke patients, is used as a method of integrating visual information through the screen [15,18,19,20]. In the virtual reality environment, visual information, proprioception, and vestibular sensory information do not synchronize, causing cybersickness, and body mechanisms necessary for movement in the real environment may not be trained [21]. To solve this problem, studies on how to match optic flow and actual movement during walking have been reported, and the results of the study proved that the synchronization of optic flow and movement induced natural walking and improved walking ability [22,23]. Lee et al. [15] reported that the speed-interactive treadmill training using smartphone-based motion tracking technology was applied to stroke patients to improve gait ability.

In this study, we applied the method used to stationary bicycle training in the previous study and verified its effectiveness. This study aimed to investigate the effects of speed-interactive pedaling training (SIPT) using smartphone-based motion-tracking technology on lower extremity function, sitting balance, and the walking ability of stroke patients.

## 2. Materials and Methods

### 2.1. Subjects

The subjects of this study were stroke patients admitted to U Rehabilitation Center in Gyeonggi Province, South Korea with the following characteristics: had chronic hemiplegia for six months or more due to stroke, able to sit for 30 min or more independently, able to walk more than 10 m using the assistive device, had the cognitive ability to participate in the study by understanding simple verbal instructions (Mini-Mental State Examination Score > 24), and had a Brunnstrom motion recovery stage of 4 or greater.

The exclusion criteria included brain injury except for stroke, orthopedic problems of the legs, such as fractures or damage to the peripheral nerves, cardiovascular diseases, vision defects, or deafness. Detailed information about the purpose and procedure of the study were explained to all subjects, and all subjects provided written informed consent for inclusion in the study. All experimental procedures in this study were approved by the Institutional Review Board of Kyungdong University.

### 2.2. Size of the Sample

This was a single-blinded, randomized study. We used a computer program (G-Power 3.19) to determine the sample size. The effect size was determined based on the sitting balance and walking speed of the pilot experiment (0.95). To calculate the effect size, we set the alpha error and power probability to 0.05 and 0.8, respectively. Therefore, we needed 19 patients per group. Twenty-one subjects per group were randomly recruited by estimating the dropout rate of approximately 10%.

### 2.3. Procedure

**Figure 1:**
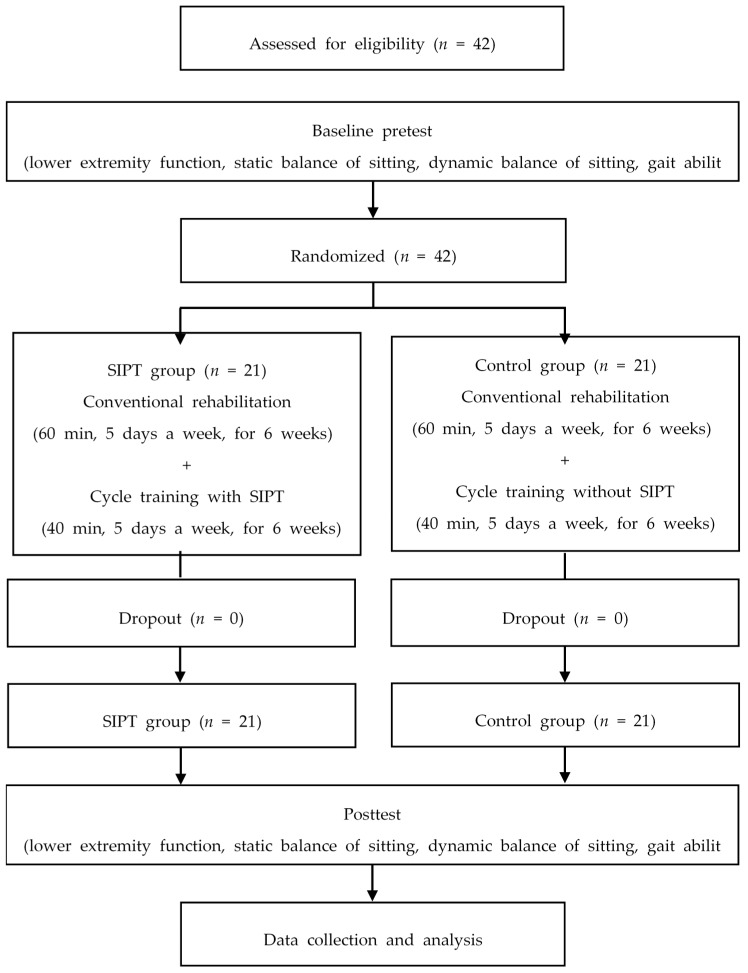
Flow diagram of the study. SIPT, speed-interactive pedaling training.

A total of 42 subjects were randomly assigned to the SIPT group (*n* = 21) and the control group (*n* = 21). Random Allocation Software was used to divide the subjects into two groups randomly. All subjects in both groups received a 60-min conventional rehabilitation program for five days per week, for six weeks. Subjects in the SIPT group used human tracking technology to perform smartphone-based pedaling training for 40 min a day, five days a week, for six weeks, and the control group performed pedaling training without SIPT for 40 min a day, five days a week, for six weeks. One week prior to training, all subjects assessed sitting balance, gait ability, and function of the lower extremity. After six weeks of training, they were evaluated in the same way as the pretest. All assessments were performed by four physiotherapists who did not participate in the training. None were dropped during the training, and the results of all 42 subjects were analyzed (Figure 1).

### 2.4. Speed-Interactive Pedaling Training (SIPT) Equipment and Mirroring Device

For SIPT, a stationary bike (MOTOmed viva, RECK-Technik, Betzenweiler, Germany), projector (BX327, LG, Seoul, South Korea), and smartphone (iPhone 8, Apple, Cupertino, California, USA) were used. A training environment was established based on previous bicycle training studies. An Apple TV (Apple, Cupertino, CA, USA) was used to project the images from the smartphone through a projector, and a High-Definition Multimedia Interface (HDMI) cable (AV10135yw4M-APL, Belkin, Playa Vista, California, USA) was used for information input and output.

### 2.5. Motion Tracking and SIPT Equipment 

**Figure 2:**
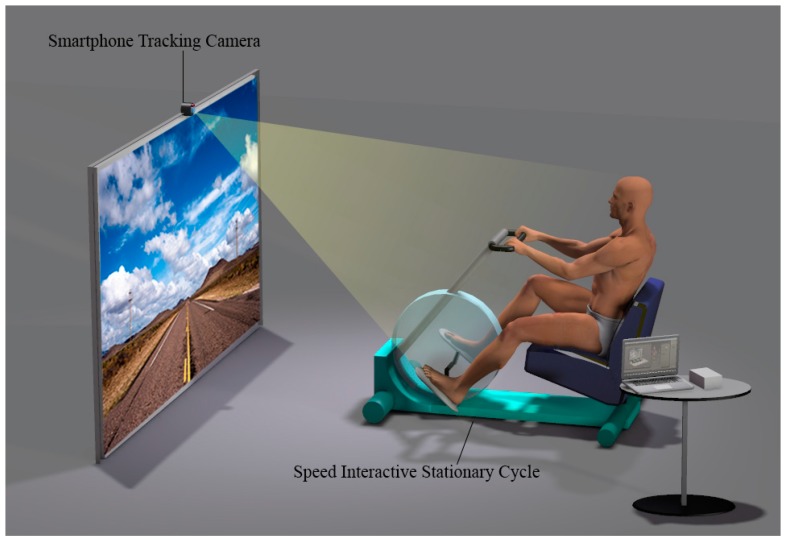
Motion tracking and speed-interactive pedaling training(SIPT) equipment.

In this study, we used a smartphone application Virtual Active (Bit Gym, Berkeley, CA, USA) for motion tracking. This application provides shooting videos of mountains, valleys, and cities that are famous tourist destinations worldwide. It consists of three modes, including walking, biking, and arm ergometer. Position the smartphone camera toward the patient to determine the patient’s pedaling speed with the smartphone camera. When the patient starts pedaling, the smartphone recognizes three virtual landmarks of the head and shoulders and draws a movement pattern. After 5 s, the pattern is recognized and the speed is synchronized with each other according to the speed of pedaling. Synchronization of the speed of optical flow, and pedaling helps the patient focus more on the virtual reality situation (Figure 2). 

### 2.6. SIPT and Pedaling Training with a Stationary Bike

We used a stationary bike for pedaling training. The bike training mode was conducted in manual mode, allowing the patient to adjust his or her own speed. The patient started at a comfortable pace that he or she normally used for training; subsequently, the pace was determined by the patient. The SIPT group pedaled while staring at the screen in front of the stationary bike, and the control group trained without the screen. Safety, while the physical therapist was training, was monitored. Patients with excessive hip abduction during cycling training had either fixed their thighs with a belt or lowered their thighs with a pedal. The training consisted of a total of 40 min, including 5 min of warm-up stretching, 5 min of slow pedaling, 25 min of the main exercise, and 5 min of cooldown. During the main exercise, each video for the SIPT group consisted of 10 min, and two 10-min videos were provided to the subjects. Depending on the patient’s condition, a break of 3–5 min was given during training. The main movement of the control group was performed in the same way as the SIPT, but no optic flow through the screen was provided. Subjects were instructed to stop at any time. The training was conducted in a secluded and quiet space.

### 2.7. Conventional Rehabilitation

The conventional rehabilitation program was conducted in the same way for both groups. It consisted of therapeutic exercise, occupational therapy, and functional electrical stimulation therapy. The therapeutic exercise was based on neurodevelopmental therapy and consisted of upper extremity exercise. Occupational therapy consisted of upper extremity functional exercises to improve the activities of everyday life. Functional electrical stimulation therapy consisted of application to the wrist extensor. Each exercise consisted of 30 min of therapeutic exercise, 20 min of occupational therapy, and 10 min of functional electrical stimulation therapy.

### 2.8. Outcome Measurements

The lower-extremity motor subscale of Fugl–Meyer Assessment (FMA-LE) was used to assess the motor recovery of the lower limb items. The FMA–LE assessment consists of 17 items. The FMA–LE motor subitems have two items measuring reflex activity, 11 items measuring synergistic movement, and three items measuring coordination. With the exception of the reflex items in both items, each item’s score is based on a three-point ordinal scale (0, not possible; 1, partial; 2, complete). The maximum score in the lower extremity for the FMA was 34. The inter-rater reliability of the lower extremity of the FMA was *r* = 0.96 [24].

In this study, a posturography system (GB300; Metitur Ltd., Jyvaskyla, Finland) was used to evaluate the static sitting balance. The system consists of an equilateral triangle force platform connected to a computer via a three-channel amplifier. The sampling frequency was 50 Hz. This equipment measures the balance between older people and patients who have had a stroke and can be used extensively. The posturography system was used to measure medial-lateral and anterior-posterior sway velocity and velocity moment of stroke patients in a sitting position. The intra-rater reliability of the posturography system used in this study was reported to have intra-class correlation coefficients (*r*) of 0.51–0.74 (front and rear speed) and 0.63–0.83 (right and left speed) [25]. To measure the static balance, the patient sat in a chair on the force platform. Patients were asked to watch a black circle at a point of 1.5 m (5 cm in diameter) for 30 s while measuring balance. The measurement was repeated three times, and the average value was used. The same procedure was repeated with the patient’s vision blocked.

The dynamic sitting balance was measured using a modified functional reach test (mFRT). The bar was fixed to the wall at the top height of the patient sitting comfortably in the chair. The subject’s hip and knee joints were bent at 90°, the chair and the popliteal area were 5 cm apart, and the feet were placed in the footrest of the chair. For the forward reach test, the shoulders were flexed as far as 90°. For the lateral reach test, the shoulders were stretched laterally as far as 90° abduction. All distance measurements were measured using a laser distance meter (Glm250vf, Bosch, Germany), and the average value was repeated three times. The inter-evaluator reliability of this test was reported as *r* = 0.97, indicating excellent reliability [26]. In this study, the Trunk Impairment Scale (TIS) was used to evaluate trunk function. The TIS consists of three subscales: static sitting posture balance, dynamic sitting posture balance, and coordination. Each subscale has 3 to 10 items. TIS scores range from a minimum of 0 to a maximum of 23. Higher scores indicate better trunk performance. The test-retest reliability of the TIS was reported as *r* = 0.96, and the inter-evaluator reliability was reported as *r* = 0.9927 [27].

Gait ability was evaluated with the gait analysis system (Optogait, Microgate, Bozen, Italy). Subjects walk at normal and comfortable speeds between two parallel transmit and receive bars. Subjects walked three times through the 5-m OptoGait pathway. The data collected included velocity, cadence, stride time, and step time as temporal gait parameters, and stride length and step length as spatial gait parameters.

### 2.9. Statistical Analysis

All data were expressed as mean and standard deviation. The Shapiro–Wilk test was used for the normality test, and all the resulting variables were found to satisfy the normality assumption. We used the paired *t*-test to compare the dependent variables within the group and the independent *t*-test and the Chi-squared test to compare the dependent variables between the two groups. Statistical significance was set to *p*-value < 0.05. Statistical Package for the Social Sciences (SPSS) version 20.0 (SPSS Inc., Chicago, IL, USA) was used for statistical analysis.

## 3. Results

Both groups participated in all stages of the experiment and participated in pretest and posttest. Therefore, a total of 42 subjects were included in the analysis, 21 of them in the SIPT group, and 21 in the control group. There was no significant difference in the general characteristics and the dependent variables between the two groups, indicating that the two groups were homogeneous before the experiment (Table 1; age, weight, height, duration of a stroke, gender, stroke type, paretic side, and cognition).

Outcome measures of lower extremity function, the static balance of sitting, the dynamic balance of sitting, and gait ability of the SIPT and control groups are shown in Table 2. At baseline, FMA, sitting balance, mFRT, TIS, and all gait variables results did not differ significantly between the intervention groups.

The changes in the lower extremity function, FMA were significantly improved from 16.91 to 19.49 in the SIPT group (*p* < 0.05), and from 17.15 to 18.59 in the control group (*p* < 0.05). However, the SIPT group showed a more significant improvement compared to the control group of the FMA (*p* < 0.05).

The changes in the static sitting balance ability, the speed of medial and lateral sway, the speed of the anterior and posterior sway, and the velocity of moment variables showed significant improvement after intervention in both groups regardless of vision (*p* < 0.05). However, the SIPT group showed a more significant improvement compared to the control group (*p* < 0.05).

The mFRT for all directions increased significantly after intervention in both groups (*p* < 0.05). In term of mFRT for forward direction, the SIPT group showed significant improvement from 302.27 mm to 328.41 mm (*p* < 0.05), and from 274.97 mm to 279.15 mm in control group (*p* < 0.05). In terms of MFRT for the non-affected side, the SIPT group showed significant improvement from 175.23 mm to 197.89 mm (*p* < 0.05) and from 158.75 mm to 161.13 mm in control group (*p* < 0.05). In terms of the mFRT for the affected side, the experimental group showed significant improvement from 88.72 mm to 108.07 mm (*p* < 0.05) and from 84.31 mm to 85.62 mm in the control group (*p* < 0.05). However, the SIPT group showed a more significant improvement compared to the control group (*p* < 0.05). In the intragroup analysis, TIS were significantly improved from 12.23 to 13.38 in the SIPT group (*p* < 0.05), and from 12.24 to 13.14 in the control group (*p* < 0.05). However, the SIPT group showed a more significant improvement compared to the control group (*p* < 0.05).

The temporal gait parameters, including velocity, cadence, Stride time, and Step time, were a significant improvement after intervention in the SIPT group (*p* < 0.05), but nothing significant in the control group. In addition, the SIPT group showed a more significant improvement compared to the control group (*p* < 0.05). The spatial gait parameters, including stride length, step length, were a significant improvement after intervention in the SIPT group (*p* < 0.05), but nothing significant in the control group. In addition, the SIPT group showed a more significant improvement compared to the control group (*p* < 0.05).

## 4. Discussion

This study aimed to investigate the effects of SIPT using the smartphone virtual reality application to improve lower extremity motor function, sitting balance, and gait in stroke patients. The control group with the stationary bicycle training was compared with the SIPT group, and the significant differences in the groups were identified.

According to the previous studies, the effects of stationary bicycle training on the recovery of stroke patients were reported to improve the function of the lower limbs, walking ability, dynamic balance, and activities of daily living, and to reduce spasticity [28].

Early exercise provided to stroke patients can lead to rapid recovery. Therefore, clinicians train stroke patients to stand and walk early [29,30]. Stationary bicycle training can provide reciprocal movement of both legs to improve lower limb movement, even in non-walking patients [7,8,9]. Stationary bicycle training is an effective exercise for the movement of the hip, knee, and ankle joints and muscles around the joints in stroke patients [7,8,9]. The movement of the joints and the activation of the muscles alone will not allow walking, but it can be a good exercise in the preliminary steps for walking [5,6].

The results of several previous studies have reported improvements in the function of the lower limbs [28]. The function of the lower extremities was mainly measured by FMA and improved after training. In this study, both groups also showed improved FMA scores. Pedaling is possible through the movement of both legs, but there is a limitation that only the movement of the unaffected side is possible [5]. The results of the FMA show that there is an improvement in the affected side, and the improved results of the SIPT group using VR means that the affected side was more actively used. In this study, we focused on improving body balance and function through pedaling training. The body requires stability and balance while pedaling. Stability and trunk balance are both prerequisites not only for pedaling but also for training purposes. Open kinetic chain movement of the legs induces reciprocal activation of the surrounding pelvic muscles. The spatiotemporal variables of gait also showed significant differences before and after training. The walking speed increased, the spatial variables also increased the length value, and the double limb support and step width decreased, indicating the stability of walking. All variables showed differences before and after training between the two groups. The difference between the two groups confirmed the effect of SIPT. However, it is difficult to explain the direct mechanisms of SIPT on the function of the lower extremities, body balance, and gait. During the training, patients in the control group exercise at a constant speed, while patients in the SIPT group experience a slow change in speed. The response of the pedaling speed to the optic flow increased the training commitment of the stroke patient and motivated the increase in speed. Studies that have applied optic flow to stroke patients have reported improvements in gait by synchronizing with the treadmill speed, and no researches have been associated with stationary bicycles [15]. Based on the results of this study, the provision of SIPT to stroke patients before entering gait training or in the stage of improving trunk stability would improve walking and postural balance and would be suitable as preparatory training for gait training. As shown in the gait training study, problems caused by inconsistency between visual and intrinsic sense can be minimized through SIPT, and various experiences could be made by experiencing the virtual space. The devices used in this study have the following advantage: the application of advanced technologies at low cost because applications used are through smartphones on existing stationary bicycles. The technology of mirroring the screen to a large screen in a smartphone is also widely available; hence, SIPT could be performed at a low cost.

This study has the following limitation: although the effects of a six-week training were verified through this study, the sustainability and the patient’s participation or motivation were not measured. In the future, the methods used in this study are expected to be used by many clinicians in therapeutic settings.

## 5. Conclusions

The results of this study showed that SIPT is a useful rehabilitation program for the functional recovery of the lower extremities of stroke patients, improving sitting balance, trunk control, and gait. Using the smartphone, the effectiveness and feasibility of training through virtual reality were shown.

## Figures and Tables

**Table 1:** General characteristics of the subjects.

	SIPT Group (*n* = 21)	Control Group (*n* = 21)	χ^2^/*t*	*p*
Age (year)	61.67 ± 8.42	64.24 ± 10.83	0.859	0.395
Height (cm)	165.62 ± 7.05	162.29 ± 9.32	1.307	0.199
Weight (kg)	62.20 ± 7.04	61.35 ± 9.15	0.334	0.740
BMI (point)	22.67 ± 2.19	23.20 ± 2.05	0.817	0.419
Duration of stroke (month)	14.81 ± 7.30	16.48 ± 7.13	0.748	0.459
MMSE	25.81 ± 1.29	25.76 ± 0.94	0.137	0.892
Gender (male/female)	14/7	13/8	0.747	0.104
Paretic side (right/left)	12/9	10/11	0.537	0.382
Stroke type (infarction/hemorrhage)	15/6	13/8	0.513	0.429

Values are expressed as mean ± standard deviation. The independent *t*-test and Chi-squared tests are used to compare the dependent variables between the two groups. SIPT, speed-interactive pedaling training; BMI, body mass index; MMSE, mini-mental state examination.

**Table 2:** Comparison of measures within groups and between groups.

Variables	SIPT Group (*n* = 21)			Control Group (*n* = 21)			Significance of Change Scores
	Baseline	Post	Change Score	Baseline	Post	Change Score	t
Lower extremity function							
FMA-LE (point)	16.91 ± 3.62	19.49 ± 3.56	2.58 ± 0.63 *	17.15 ± 3.13	18.59 ± 2.72	1.44 ± 2.41 *	2.347 ^†^
Static sitting balance ability							
EO-MLS (mm/s)	3.95 ± 1.27	3.00 ± 0.82	−0.95 ± 0.88 *	3.75 ± 1.21	3.34 ± 1.03	−0.41 ± 0.92 *	2.461 ^†^
EO-APS (mm/s)	5.85 ± 1.41	4.60 ± 1.38	−1.25 ± 0.81 *	5.89 ± 1.18	5.20 ± 1.30	−0.69 ± 0.64 *	2.282 ^†^
EO-VM (mm/s^2^)	5.06 ± 2.18	3.71 ± 1.68	−1.35 ± 0.97 *	4.74 ± 2.04	4.12 ± 1.93	−0.62 ± 1.07 *	2.313 ^†^
EC-MLS (mm/s)	3.95 ± 1.27	2.85 ± 0.66	−1.10 ± 0.98 *	3.75 ± 1.21	3.24 ± 0.93	−0.50 ± 0.84 *	2.098 ^†^
EC-APS (mm/s)	5.85 ± 1.41	4.73 ± 1.43	−1.12 ± 0.71 *	5.89 ± 1.18	5.20 ± 1.30	−0.69 ± 0.64 *	2.059 ^†^
EC-VM (mm/s^2^)	4.18 ± 1.30	2.79 ± 1.32	−1.39 ± 0.76 *	4.03 ± 1.13	3.27 ± 1.29	−0.76 ± 1.06 *	2.227 ^†^
Dynamic sitting balance ability							
mFRT-forward (mm)	302.27 ± 113.40	328.41 ± 108.52	26.14 ± 22.12 *	274.97 ± 122.87	279.15 ± 126.13	4.18 ± 6.11 *	4.384 ^†^
mFRT-non-affected (mm)	175.23 ± 48.60	197.89 ± 54.79	22.66 ± 20.57 *	158.75 ± 61.74	161.13 ± 63.61	2.38 ± 5.07 *	4.388 ^†^
mFRT-affected (mm)	88.72 ± 24.24	108.07 ± 33.26	19.35 ± 14.96 *	84.31 ± 37.48	85.62 ± 38.88	1.68 ± 3.07 *	5.302 ^†^
TIS (score)	12.33 ± 1.59	14.38 ± 2.09	2.05 ± 1.20 *	12.24 ± 1.89	13.14 ± 0.48	0.90 ± 1.70 *	2.515 ^†^
Gait ability							
Temporal gait parameter							
Velocity (cm/s)	0.46 ± 0.15	0.56 ± 0.18	0.10 ± 0.04 *	0.41 ± 0.22	0.42 ± 0.22	0.01 ± 0.03	8.135 ^†^
Cadence (step/min)	76.26 ± 14.43	83.34 ± 16.11	7.08 ± 3.38 *	74.56 ± 15.77	75.40 ± 18.05	0.84 ± 4.09	5.389 ^†^
Stride time (sec)	1.63 ± 0.30	1.49 ± 0.28	−0.13 ± 0.07 *	1.67 ± 0.29	1.67 ± 0.33	0.00 ± 0.09	5.427 ^†^
Step time (sec)	0.81 ± 0.15	0.74 ± 0.13	−0.07 ± 0.04 *	0.83 ± 0.15	0.83 ± 0.17	0.00 ± 0.04	5.247 ^†^
Spatial parameter							
Stride length (cm)	71.66 ± 18.57	80.44 ± 20.00	8.77 ± 3.75 *	63.59 ± 21.67	65.63 ± 20.97	2.04 ± 5.81	4.461 ^†^
Step length (cm)	35.87 ± 9.22	40.24 ± 9.96	4.37 ± 1.88 *	31.61 ± 10.40	32.82 ± 10.48	1.20 ± 3.09	4.016 ^†^

Values are expressed as mean ± standard deviation. ^*^ means significant difference within group. ^†^ means significant difference between group. SIPT, speed-interactive pedaling training; FMA-LE, lower-extremity motor subscale of Fugl-Meyer Assessment; EO—eye opened; EC—eye closed; MLS—medial-lateral speed; APS—anterior-posterior speed; VM—velocity moment; mFRT, modified functional reach test; TIS, trunk impairment scale
